# Using Hybrid Nanoplatforms to Combine Traditional Anti-Inflammatory Drug Delivery with RNA-Based Therapeutics for Macrophage Reprograming

**DOI:** 10.3390/ijms251910693

**Published:** 2024-10-04

**Authors:** Ana F. Almeida, Margarida S. Miranda, Rui L. Reis, Manuela E. Gomes, Márcia T. Rodrigues

**Affiliations:** 1 3B’s Research Group, I3Bs—Research Institute on Biomaterials, Biodegradables and Biomimetics, University of Minho, Headquarters of the European Institute of Excellence on Tissue Engineering and Regenerative Medicine, AvePark, Parque de Ciência e Tecnologia, Zona Industrial da Gandra, 4805-017 Barco, Guimarães, Portugal; ana.almeida@i3bs.uminho.pt (A.F.A.); margarida.miranda@i3bs.uminho.pt (M.S.M.); rgreis@i3bs.uminho.pt (R.L.R.); 2 ICVS/3B’s–PT Government Associate Laboratory, 4710-057 Braga, Guimarães, Portugal

**Keywords:** magnetic micelles, precision technologies, drug delivery, celecoxib, anti-inflammatory drug, miR, RNA-based technologies, inflammation

## Abstract

There is growing evidence on the significant role of prolonged inflammation in triggering and progressing of numerous diseases with substantial health and socioeconomic impacts, such as musculoskeletal, cardiovascular and autoimmune disorders, and cancer. Therefore, there is an urgent need to develop therapies that can overcome the main challenges of currently used approaches, such as non-target action, partial modulation of the complex inflammatory pathways, and short-term effects, to effectively manage and resolve chronic inflammatory states. This work investigates the therapeutic synergy of clinically relevant anti-inflammatory agents approaching naïve and classically activated macrophages owing to their central role in inflammation. Aiming at human therapies, a dual-loading nanoplatform reunites molecules with different physico-chemical properties in a single system, seeking to more effectively and comprehensively regulate macrophage functions for precision cell guidance and greater versatility in disease managing. To build this platform, palmitic acid *grafted* chitosan, superparamagnetic iron oxide nanoparticles, the clinically approved NSAID celecoxib (also known as Celebrex^®^), and RNA technologies were combined into superparamagnetic polymeric micelles (SPMs). Our findings demonstrated that traditional anti-inflammatory drugs such as celecoxib and microRNA molecules were efficiently delivered by the SPMs, altering the inflammatory profile of naïve (M0φ) and M1-primed macrophages (M1φ) assessed by gene and protein expression. The impact of the dual-loaded SPMs in naïve Mφ is an interesting finding towards the modulation of the initial immune response, reducing the potential for chronic inflammation and promoting tissue healing. Collectively, these encouraging results demonstrate the promise of multi-nanomedicine strategies to enhance the efficacy of therapeutic interventions by offering a fresh approach to more precisely and carefully regulated nanotherapeutics delivery.

## 1. Introduction

Long-term inflammation is a key factor in the pathology of most chronic diseases including musculoskeletal [1,2], cardiovascular or auto-immune disorders and cancer [3,4,5], which stand as the leading causes of death and disability worldwide.

Conventional treatments for chronic inflammation aim to reduce the inflammatory response, alleviate symptoms, and prevent further tissue damage. These treatments include lifestyle changes, physical therapies, pharmacological interventions, and, in some cases, surgical options [6] depending on the specific condition, severity of inflammation, patient characteristics, and response to therapy.

Pharmaceutical drugs such as nonsteroidal anti-inflammatory drugs (NSAIDs) are often the first line for managing mild to moderate inflammation in musculoskeletal injuries and related pathologies [7,8] due to their relatively rapid onset of action. While very effective against inflammation signs and lessening pain and discomfort, anti-inflammatory drugs do not treat tissue damage. Moreover, their lack of specificity and non-targeted nature results in limited efficacy, necessitating multiple- and/or high-dose requirements, and adverse outcomes [7], including cytotoxicity and drug resistance, with relevant implications on patients’ health. To control more severe inflammatory conditions, corticosteroids and disease-modifying agents are used as secondary therapies [9,10]. Disease modifying agents are highly specific in their target action, but like steroids, they also have immunosuppressive effects. This can result in notable side effects, including bone marrow suppression, liver toxicity (hepatotoxicity), and a higher risk of infections [11].

The approval of Food and Drug Administration (FDA) of RNAi medications and those siRNAs and miRNA-based therapeutics entering into clinical investigations [12] has paved the way for a new generation of RNA-based medicines, contributing to innovative therapeutic options and expanding the possibilities for managing chronic inflammation diseases. Within the RNA superfamily, the microRNAs (miRs), offer the regulation of multiple genes and pathways simultaneously, providing broader yet more precise molecular routes than traditional drugs. miR binds to specific sequences at the 3′ UTR of their target mRNA molecules, which either causes translational repression or the target mRNA molecule to degrade. miR dysregulation has been implicated in complex inflammatory disorders, including rheumatoid arthritis, inflammatory bowel disease and arteriosclerosis [12]. Some miR can influence the inflammatory status of cells and fine-tuning of the immune response, reducing deleterious mediators as inflammatory cytokines while preserving or enhancing the pro-healing functions of immune cells.

The complementary use of conventional drugs and RNA therapeutics ventures a more holistic approach, likely supporting the transition from a pro- to a resolving inflammation state, aiding in tissue functional recovery, and paving the way for enhancing treatment efficacy and patient outcomes. Combining several medicines in one nanocarrier boosts effectiveness, minimizing systemic exposure and side effects associated with high dosages and drug resistance. Moreover, multi-modal systems simplify both the administration and treatment regimen, improving patient compliance and convenience.

Thus, this work aims to investigate the influence of combined delivery of drugs (celecoxib) and miR molecules (miR155 antagonist), leveraging the unique regulatory capabilities of both medicines, in the functional responses of naïve and pro-inflammatory (M1φ) macrophages, potentially leading to better disease management.

Cyclooxygenase-2(COX2) is an enzyme produced by macrophages (Mφ) and other cells with a crucial role in the inflammatory process by converting arachidonic acid into prostaglandins. Prostaglandins amplify and prolong the inflammation, recruiting immune cells and sustaining the M1 phenotype. As a selective COX2 inhibitor, celecoxib (CCXB) is approved for some types of musculoskeletal disorders [13,14] and has shown potential in cancer chemoprevention and therapy [15,16]. CCXB is also noted for its reduced risk of gastrointestinal bleeding compared to other NSAIDs [17]. In an interdependent manner, the delivery of an miR antagonist to miR155, which is upregulated in response to inflammatory stimuli such as lipopolysaccharides (LPS), interferon-gamma (IFNγ), and tumor necrosis factor-alpha (TNFα) could reduce excessive M1 polarization. Likewise, the inhibition of miR155 could change Mφ activities and regulatory functions, associated with inflammatory pathways such as nuclear factor-kappa B (NFκB) and JAK/STAT, contributing to decreasing the production of inflammatory cytokines such as interleukin-6, -1 beta, -8 (IL6, IL1β and IL8), TNFα, and enzymes such as COX2.

Both medicines were incorporated within superparamagnetic polymeric micelles (SPMs) assembled from palmitic acid-*grafted*-chitosan (PA-*g*-CHI) and superparamagnetic iron oxide nanoparticles (SPIONs). Due to their distinct properties and solubility profiles, SPMs allow hydrophobic CCXB loads in its core, and hydrophilic miR on its shell. PA is resourceful for its hydrophobic properties while CHI is a polysaccharide derived from chitin, and is biocompatible and positively charged, which is highly relevant for the association of miR to the shell. Besides the natural-origin materials that confer inherent compatibility of SPMs with biological systems, the polymeric micellar nanostructure offers high stability, protection, and high-loading rates of lipophilic CCXB, enabling the polyelectrolyte interactions between miR and CHI at the shell.

Additionally, SPIONs provide magnetic sensitivity and responsiveness upon magnetic field actuation, being widely used in biomedical and clinical applications [18,19]. Within our platform, SPIONs not only consider utilizing magnetofection, an efficient technology for cell uptake surmounting cell membrane barriers, but also anticipate the benefit of imaging and control of SPMs locally with potential for adjusting a patient’s dosages through magnetically assisted release of cargos [20]. Gathering the advantages of polymeric micelles and inorganic magnetic nanoparticles, our hybrid system will simultaneously carry drugs and miR with interrelated actions over inflammatory triggers, overcoming the limitations on efficient delivery to target cells and cellular uptake of currently nanoparticulate-based systems.

In past studies, we have delved into in vitro cell guidance, specifically addressing inflammation regulation and impaired healing using SPION-based systems and miR-based technologies. These systems displayed pro-regenerative stimulation by showing control over macrophage responses [21] and in inflammatory-primed cells [22]. Our team has been further developing chimeric particles to create multi-modal systems recuring to model drug candidates. Nevertheless, we have not investigated anti-inflammatory drugs or the synergy of drugs with miR using SPM systems nor its potential applicability to naïve cells. Thus, in this work, we are taking a significant step forward by investigating an anti-inflammatory drug/miR dual strategy with clinical significance in immune cell populations with central roles in the pathophysiological events leading to chronic inflammation.

Naïve macrophages (M0φ) provide a flexible target and novel opportunities to modulate immune responses, and consequently, the inflammatory milieu at earlier stages. Early intervention targeting M0φ could be a game changer in preventing the progression of chronic inflammation diseases, by stimulating M0φ to adopt pro-reparative phenotypes as they reach and accumulate in the inflammation zone. This versatility is also valuable for developing personalized medicine approaches.

Our results showed an efficient dual delivery strategy of CCXB and miR, with sustainable loading and release of the drug at different pH and structural stabilities in aqueous solutions, including physiologically relevant solutions. The delivery of both cargos led to changes in the inflammatory profile of Mφ in different phenotypic states. These promising findings highlight the potential of multi-medicine, particle-based nanoplatforms to advance the treatment of chronic inflammatory diseases, providing a new avenue for achieving better clinical outcomes through precision delivery.

## 2. Results and Discussion

### 2.1. Loading of Medicines in Superparamagnetic Micelles (SPMs)

**Figure 1:**
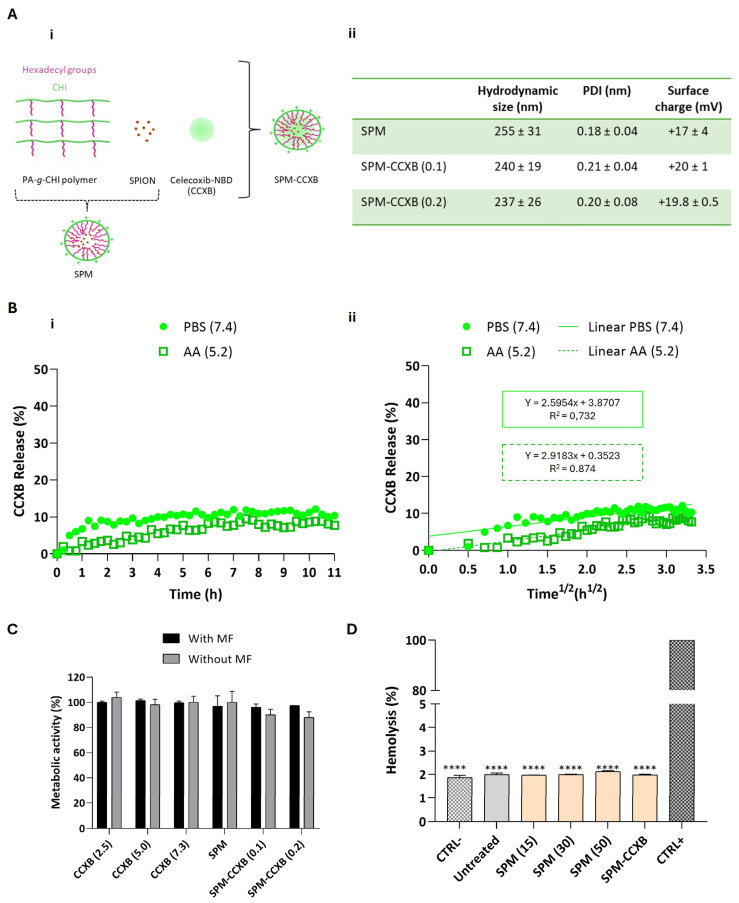
Determination of the stability, release profiles and biocompatibility of SPM-CCXB. (**A**) (i) Schematic representation of the fabrication of hybrid superparamagnetic micelles with celecoxib NBD (SPM-CCXB). Owing to its hydrophobic properties, celecoxib-NBD (green) is stored in the core of SPM; (ii) hydrodynamic size, polydispersity index (PDI) and surface charge of CCXB-free SPM (SPM) and SPM loaded with two SPION:PA-g-CHI:CCXB mass ratios: 1:5:0.1 and 1:5:0.2, SPM-CCXB (0.1) and SPM-CCXB (0.2), respectively). (**B**) (i) Release profile of CCXB from SPM-CCXB in function of time in PBS (pH 7.4) and acetic acid (AA, pH 5.2); (ii) CCXB release behavior best fitted to the Higuchi model (percentage of drug released vs. square root of time) at PBS 7.4 and AA 5.2, with correlation coefficient values (*R*^2^). (**C**) Assessment of cell viability on macrophages, after treatment with free-form CCXB at different concentrations (2.5, 5.0, and 7.3 µg/mL, which correspond to 4.3, 8.6, and 12.4 µM, respectively), CCXB-free SPM and different formulations of SPM-CCXB with and without the use of a magnetic field (MF). Untreated cells were used as the positive control of cell viability (=100%) using the PrestoBlue method. (**D**) Hemolysis assay performed with mild agitation after incubation with CCXB-free SPM at different concentrations (15, 30, and 50 µg/mL), SPM-CCXB (0.1) and SPM-CCXB (0.2). Data were analyzed against SDS, the positive control (CTRL+) by the One-Way ANOVA, followed by the Dunnett test (**** *p* < 0.0001). Data represent the mean ± SD of three independent experiments.

The dual-medicine loads were enabled by the production of micelles in two sequential stages. Firstly, palmitic acid *grafted* chitosan, superparamagnetic iron oxide nanoparticles, and CCXB were self-assembled into nanoparticles shielding the drug cargo (SPM-CCXB) and are schematically presented in Figure 1A(i).

To validate our system for pharmacological applications, we selected and investigated a fluorophore-labeled celecoxib, CCXB-NBD (referred as CCXB in the experimental studies), where celecoxib is bonded to the fluorescent molecule 7-nitrobenzofurazan (NBD) [23], thus facilitating the measurements of drug loading and release, and detection of cell uptake. At a second stage, the CCXB-loaded SPMs with the positively charged CHI shell was electrostatically bonded to the negatively charged miR molecules (SPM-CCXB/miR) forming the dual-loading platform. This two-step procedure was necessary to avoid organic solvents and chemical modifications that could disturb the structural stability and biofunctionality of miR molecules and compromise the molecular recognition and interactions with cell endogenous machinery.

Prior to analyzing the synergistic effects of anti-inflammatory medicines on macrophage responses, we thoroughly studied the stability, loading, and release profiles of CCXB cargo and the cyto- and hemo-compatibilities of miR, and their inherent impact on the system, which is the main novelty of this work.

### 2.2. Assessment of Structural Stability of the Drug-Loaded SPMs (SPM-CCXB)

Celecoxib-NBD-loaded SPMs (SPM-CCXB) were prepared from the amphiphilic polymer PA-*g*-CHI, SPIONs, and CCXB by an ultrasonication treatment using two different SPION:PA-*g*-CHI:CCXB mass ratios: 1:5:0.1 and 1:5:0.2. Two amounts of CCXB were investigated to achieve a high level of drug load in the core of the SPM. The two formulations, SPM-CCXB (0.1) and SPM-CCXB (0.2), were characterized by dynamic light scattering (DLS) and electrophoretic light scattering (ELS), and the results are presented in Figure 1A(ii). We have found no significative difference among the size of SPM-based formulations. Moreover, the CCXB amounts investigated did not influence the dimensions of the system nor the size distribution, which can be explained by their successful incorporation inside the SPM, and to the stability of the shell. The nanoparticles have a uniform size distribution (Figure 1A(ii)) with evidence of colloidal stability in acidic aqueous suspension for at least 15 days when stored at 4 °C (Supplementary Information, Figure S1). Moreover, both SPM and SPM-CCXB present a positive zeta potential due to the cationic charge of the amino groups of chitosan present in the shell of the nanoparticles.

Since SPM-CCXB (0.1) and SPM-CCXB (0.2) present similar characterization results in terms of size, polydispersity index (PDI), and surface charge, we decided to continue the studies with the SPM with a higher drug amount.

### 2.3. Determination of SPION Content, and Loading and Release Profile of Celecoxib in SPM-CCXB

SPIONs and CCXB were included in the core of the polymeric micelles. The SPION-loading efficiency (LE) and loading content (LC) of the SPM were determined from inductively coupled plasma-optical emission spectroscopy (ICP-OES) measurements of the iron concentration in the core of SPMs and SPM-CCXB (Table 1). To determine SPM potential as an anti-inflammatory drug delivery nanocarrier, the LE and LC of CCXB was determined from fluorescence measurements.

Equations (1) and (2) were used to calculate the LE and LC, respectively, of both the SPIONs and the drug and the results are presented in Table 1.

(1) $$\mathrm{LE}\;\left(\%\right)=\frac{\mathrm{mass}\;\mathrm{of}\;\mathrm{drug}\;\mathrm{or}\;\mathrm{SPION}\;\mathrm{in}\;\mathrm{micelles}}{\mathrm{mass}\;\mathrm{of}\;\mathrm{drug}\;\mathrm{or}\;\mathrm{SPION}\;\mathrm{in}\;\mathrm{feeding}}\times \;100$$

(2) $$\mathrm{LC}\;\left(\%\right)=\frac{\mathrm{mass}\;\mathrm{of}\;\mathrm{drug}\;\mathrm{or}\;\mathrm{SPION}\;\mathrm{in}\;\mathrm{micelles}}{\mathrm{mass}\;\mathrm{of}\;\mathrm{drug}\;\mathrm{or}\;\mathrm{SPION}-\mathrm{loaded}\;\mathrm{micelles}}\times \;100$$

The LE of SPIONs for SPM-CCXB does not significantly differ from that of SPMs, suggesting that CCXB does not affect SPION incorporation in the system. The SPION content falls within the range observed for other SPM formulations also incorporating hydrophobic molecules: 3–33% [19]. Moreover, the LC of CCXB for SPM-CCXB is similar to other SPM formulations incorporating drugs with similar molecular weight such as doxorubicin (6–12%) [24,25,26,27] and tamoxifen (8.19%) [28].

The release behavior of CCXB from the SPM was investigated at pH 7.4 to simulate the physiological conditions and at pH 5.2 to mimic acidic intracellular compartments. For that, we have incubated SPM-CCXB in a phosphate-buffered saline (PBS) pH 7.4 solution and an acetic acid (AA) 20 mM pH 5.2 solution and followed the fluorescence intensity of the nanoparticles over time in both solutions. In Figure 1B(i), the percentage of released CCXB from SPMs, in each solution as a function of time, is shown. At both pHs, the release of CCXB to the aqueous medium is sustained, reaching a ~10% release after 11 h. This behavior can be attributed to the hydrophobic nature of CCXB, which leads to gradual and slow release in aqueous media. Other works reported in the literature present similar behaviors. For example, in the work by Sabra et al. [29], dasatinib (DAS) was incorporated in zein-lactoferrin superparamagnetic micelles and the micelles exhibited a very slow release of DAS without an initial burst at pH 7.4. About 5.21% of the drug was released in the first 4 h and then a sustained drug release profile was achieved in which 15.36% of the drug was released after 120 h. The release percentage of CCXB is not significantly dependent on pH being slightly higher at pH 7.4 compared to pH 5.2.

We have further analyzed the release behavior of CCXB using different dissolution models including zero-order, first-order, and Higuchi and Korsmeyer–Peppas models. The model that best fitted the CCXB release was the simplified Higuchi model represented by the following equation:

*Q* = K_H_*t*^1/2^ (3)

where the amount of drug released (*Q*) is proportional to the square root of time (*t*) and K_H_ is the release kinetics constant of Higuchi (Figure 1B(ii)). For PBS 7.4, the K_H_ was 2.6 h^−1/2^ and the correlation coefficient factor *R*^2^ = 0.732, whereas for AA 5.2, the K_H_ was 2.9 h^−1/2^ and the correlation coefficient factor *R*^2^ = 0.874. The good fitting to the Higuchi model indicates a drug release controlled by diffusion.

The initial release of the CCXB is both low and desirable, featuring an effective sustained release mechanism. Although a small portion of the drug (~10%) may be lost before reaching the inflammation zone, the release rate remains stable over time, ensuring that the particle and CCXB reach the target site where the therapeutic effect is most needed. The controlled release of CCXB offered by this system is supported by the colloidal stability data of SPM formulations and represents a significant improvement over conventional drug delivery nanosystems. It ensures a more consistent and sustained delivery of the hydrophobic cargo intended for these particles, reducing the risk of initial burst release and high exposure to drugs, and enhancing the overall efficacy and safety of the treatment.

### 2.4. Evaluation of Biological Compatibility of SPM-CCXB

With the exception of SPIONs, the materials used in SPM assembly share a natural origin, and consequently, they are more likely to be recognized by the body as non-hazardous, reducing the likelihood of triggering exacerbated immune responses. Additionally, their biodegradable nature facilitates their clearance from the body after delivery, thereby minimizing the risk of bioaccumulation and potential toxicity for the patient. Nevertheless, and due to the lack of information concerning CCXB dosages in vitro, in particular celecoxib conjugated with NBD fluorescence probe, we investigated different concentrations of free CCXB to determine their safe use on cultured cells (Figure 1C). The selection of these concentrations was guided by literature references [23,30] and was carefully considered to avoid overdosing, which could compromise cell viability and hinder the study of the drug effects on cell processes. Cells exhibited high-metabolic activity levels in the presence of the drug concentrations assessed, either as free forms or being SPM loaded, suggesting the adequacy of celecoxib concentrations and the use of NBD-conjugated celecoxib to pursue the SPM-based studies. The outcomes are also in accordance with reported studies [23,30,31].

One of the major goals for including SPIONs is enabling magnetofection. Magnetofection is a highly efficient cell uptake technology based on a magnetic field (MF) exerted on SPIONs that draws associated nucleic acids towards cells [32]. Compared to other non-magnetic procedures such as lipid or polymer-based transfection vectors, magnetofection evidences higher internalization efficiency and a shorter delivery time [33]. In previous studies, we have reported the cytocompatibility features of magnetically assisted internalization of SPION-based systems such as magnetoplexes [21], and cell sheets [34,35] in various cell types and co-culture models. Concordantly to these works, the viability and metabolic activity rates of cells internalized with SPM-CCXB are close to untreated cells, evidencing that the actuation of an external MF to enable magnetofection is cell compatible either in free form of CCXB or loaded in SPMs.

Envisioning the clinical application, SPM systems will contact and likely circulate in biological fluids. Thus, in vitro determination of hemolytic properties is important for the preliminary evaluation of SPM compatibility in blood. The low values detected in both SPM and SPM-CCXB conditions (<2%, *p* < 0.0001 in comparison to the positive control) do not correlate to hemolysis in human red blood cells (Figure 1D), which strengthen the use of SPMs in therapeutics.

### 2.5. Fluorescence Spectra and Cell Uptake Assessment of SPM-CCXB

**Figure 2:**
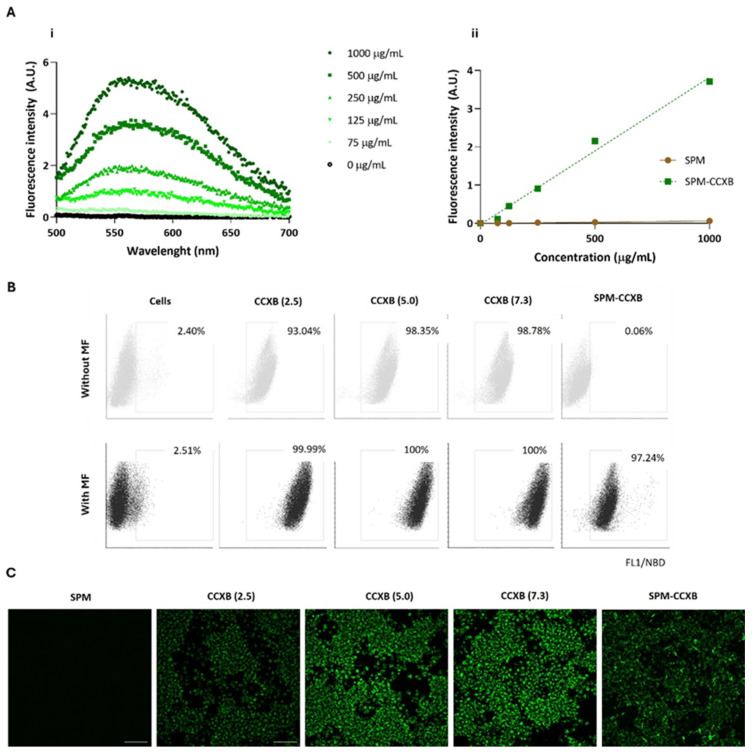
Fluorescence spectra and cell uptake evaluation of celecoxib in its free form (CCXB) and loaded into SPM (SPM-CCXB). (**A**) Fluorescence emission spectra of SPM-CCXB (i) at different drug concentrations; (ii) fluorescence intensity represented as a function of concentration and a linear correlation was obtained (*R*^2^ = 0.990). (**B**) Flow cytometric analysis of CCXB (2.5, 5.0, and 7.3 µg/mL, which correspond to 4.3, 8.6, and 12.4 µM, respectively) and SPM-CCXB uptake from macrophages (Mφ) assisted (with MF) or not (without MF) by magnetofection (MF). (**C**) Fluorescence microscopy images representative of colocalization of CCXB and SPM-CCXB in Mφ assisted by magnetofection. Scale bar = 100 µm.

The fluorescence emission spectra of the SPM-CCXB were obtained using nanoparticle suspensions with concentrations from 0 to 1000 µg/mL and with an excitation wavelength of 471 nm. The emission spectra presented in Figure 2A(i) show a concentration-dependent fluorescence increase for the SPM-CCXB. The fluorescence intensity of the SPM-CCXB (λ_exc_ = 471 nm and λ_em_ = 586 nm) was represented as function of concentration in Figure 2A(ii) and a linear correlation was obtained allowing for the concentration of the nanoparticles to be estimated.

The cytometry data showed that Mφ can easily internalize free drugs within the concentration range investigated as shown by the high percentage of the positive cell populations (>93%) in comparison to the untreated/unstained cells (<3%) (Figure 2B). Due to its small size and lipophilic nature, CCXB is likely to primarily enter cells through passive diffusion across the lipid bilayer of cell membranes. Passive diffusion is a common mechanism for the cellular uptake of lipophilic drugs that does not require energy and occurs down the concentration gradient of the drug. This effect is likely supported by the small dimension of the drugs and to the pharmacological activities of NSAIDs on membrane permeabilization activity [36].

The presence of an MF mediated by a magnetofection protocol favors an increase of 6.5% of positive cells, and thus a more efficient internalization of free-form drugs at lower concentrations (2.5 µg/mL). Independently of drug concentration, MF stimulation resulted in the successful uptake of CCXB by the entire population of cells.

The results also indicated that MF actuation is critical for SPM-CCXB detection within the Mφ. The percentage of positive cells for NBD available in SPM-CCXB goes from 0.06% to 97% after MF stimuli, which confirms magnetofection as a high-efficacy method for cellular uptake and the suitability of SPM to effectively carry a therapeutic cargo into living cells. Interestingly, the magnetofection seems to influence the fluorescent intensity of the free drug in a concentration-dependent manner. This behavior is not so evident in drug-loaded SPM, which may be explained by the protective coating effect of SPM shell. In vitro uptake of free drug and SPM-CCXB was also confirmed by detecting the fluorescence signal emitted by NBD fluorophore in Mφ previously incubated with CCXB and SPM-CCXB. As shown in Figure 2C, the cells displayed green fluorescence in the cytosol, which indicates the presence of SPMs inside the cells and successful delivery of CCXB.

### 2.6. Confirming the Cyto- and Hemo- Compatibility of SPM-miR

Several miRs were identified in Mφ polarization and in the regulation of inflammatory responses [37]. In particular, miR155 is a crucial regulator of inflammation, influencing both the initiation and resolution of immune responses. An apolipoprotein E (ApoE)/miR155 double knockout (ApoE^−/−^ miR155^−/−^) mice model has shown a decreased Mφ inflammation and reduced atherosclerotic lesion development [38], pointing to cell guidance competencies of miR155 in leukocytes. As a potential target aimed at controlling excessive inflammation, decreasing endogenous miR155 levels using an miR155 antagonist is important for inflammation resolution and to balance inflammatory triggers towards less inflammatory states [39]. This shift is essential for adequately regulating the ongoing inflammation process and preventing the perpetuation of chronic inflammation stimuli.

**Figure 3:**
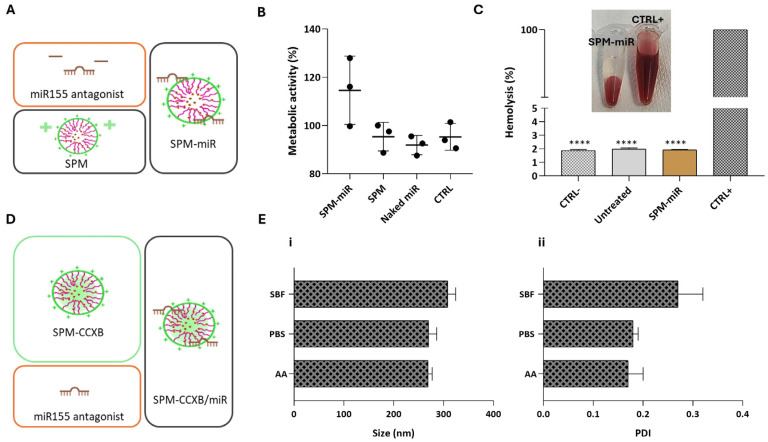
Assessment of biological compatible features of SPM-miR and structural stability of SPM-CCXB/miR in different aqueous solutions. (**A**) Schematic representation of the fabrication of SPMs with an miR155 antagonist, an inhibitor of pro-inflammatory miR155 (SPM-miR). (**B**) Assessment of Mφ viability 24 h after treatment with SPM-miR, SPMs and Naked miR (free-form of miR). (**C**) Hemolysis assay performed with mild agitation after incubation with SPM-miR. Data were analyzed against SDS, the positive control (CTRL+) by the One-Way ANOVA, followed by the Dunnett test (**** *p* < 0.0001). (**D**) Schematic representation of the fabrication of SPMs with CCXB and miR (SPM-CCXB/miR). (**E**) (i) and (ii) colloidal stability of SPM-CCXB/miR in simulated body fluid (SBF), PBS, and acetic acid (AA) solutions. Data represent the mean ± SD of three independent experiments.

We have previously investigated the influence of the miR155 antagonist in M1φ using magnetoplexes formed by electrostatic interactions between miR and SPIONs [21]. Magnetoplexes successfully induced miR-mediated gene silencing by enhancing the *IL4* and *IL10* expression, increasing the number of CD206^+^ macrophages and the IL4 concentration. Nevertheless, and to confirm the biologically compatible features of SPM-miR in Mφ (Figure 3A), we performed viability and blood compatibility assays. As expected, SPM-miR showed no negative effects on Mφ metabolic activity (Figure 3B). Similarly to SPM-CCXB, the SPM-miR showed non-hemolytic properties (Figure 3C) supporting their potential for dual payload use in medical applications.

### 2.7. Determination of SPM-CCXB/miR Stability in Aquous Solutions with Physiological Relevancy

The following step was to fabricate SPM-CCXB/miR nanoplatforms as schematically represented in Figure 3D and determine their stability in physiologically compatible solutions and acidic environments.

The size and PDI of SPM-CCXB/miR were shown to be stable at different solutions (Figure 3E). In PBS, the size and PDI are like those in an acidic solution (AA). On the other hand, these parameters seem to be slightly increased in simulated body fluid (SBF) which may be due to the higher number of different salts in this solution.

### 2.8. Determination of Macrophage Profiling and Interactions with SPM-CCXB/miR

**Figure 4:**
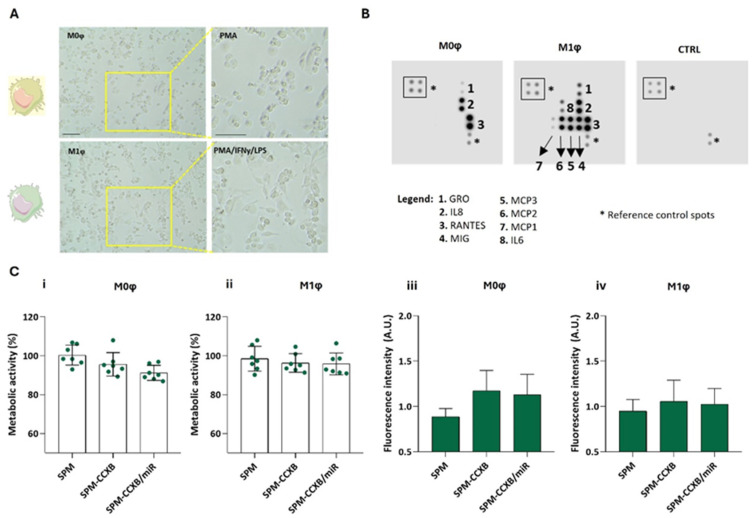
Differential profiling of Mφ. (**A**) Morphological features of naïve M0φ (stimulated PMA) and M1φ (stimulated PMA followed by treatment with IFNy and LPS). Scale bar = 100 µm. (**B**) Blot analysis identifying distinctive molecules produced by M0φ and M1φ. A membrane incubated with cell culture medium was used as control (CTRL). (**C**) Metabolic assessment and fluorescence intensity measurement of (i,iii) M0φ and (ii,iv) M1φ treated with SPM-based systems: cargo free (SPM), single loaded (SPM-CCXB), and dual-loaded SPMs (SPM-CCXB/miR) for 20 min with magnetofection. Untreated cells were used as positive control in the metabolic assessment experiments. Data represent the mean ± SD of at least three independent experiments.

Depending on the microenvironmental stimuli Mφ may go from a naïve (M0φ) to an inflammatory state (M1φ) which can be identified by morphological changes and by assessing cytokine production. M1φ differentiation from M0φ in vitro is well described in the literature involving IFNy and LPS treatment [40]. Naïve Mφ are typically round, small, with few pseudopodia, whereas M1-primed Mφ usually present an elongated and irregular shape, with extended pseudopodia and larger cytoplasm volume (Figure 4A) [41].

As observed in Figure 4B, the released cytokines are also distinguishable between the Mφ states. M0φ was characterized for a low amount of cytokine production, whereas M1φ for an elevated production of cytokines/chemokines such as growth-regulated protein (GRO, also known as CXCL1), monokine induced by INFγ (MIG, also known as CXCL9), monocyte chemoattractant protein (MCP)-1, 2, and 3, and IL6. IFNγ and LPS stimuli lead to the activation of transcription factors like nuclear factor-κB (NFκB), which in turn increase the expression of various chemokines, including GRO and MIG [42,43]. Unlike M0φ, M1-activated Mφ produce MCP-1, 2, and 3, which are crucial for recruiting monocytes, T cells, and other immune cells to the site of inflammation ensuring that the immune system can effectively deal with tissue damage [44]. Concordantly, IL6 is increased in inflammatory Mφ supporting IL6 participation in M1φ polarization [45]. In sum, the blot analysis validates the protocol used to differentiate M0φ from M1φ states, resulting in the activation of specific signaling pathways and transcription factors that drive the expression of these chemokines in response to inflammatory stimuli.

Moreover, as expected, the dual-loading nanoplatform does not compromise neither M0φ (Figure 4C(i)) or M1φ (Figure 4C(ii)) metabolic activity, and the photometric measurements of the cells cultured with the single- or dual-loaded nanoplatforms indicate higher fluorescence intensity in comparison to non-loaded SPMs (Figure 4C(iii,iv)), supporting the internalization of the SPM independently of the Mφ state.

### 2.9. Macrophages Inflammatory Performance upon SPM-CCXB/miR Treatment

**Figure 5:**
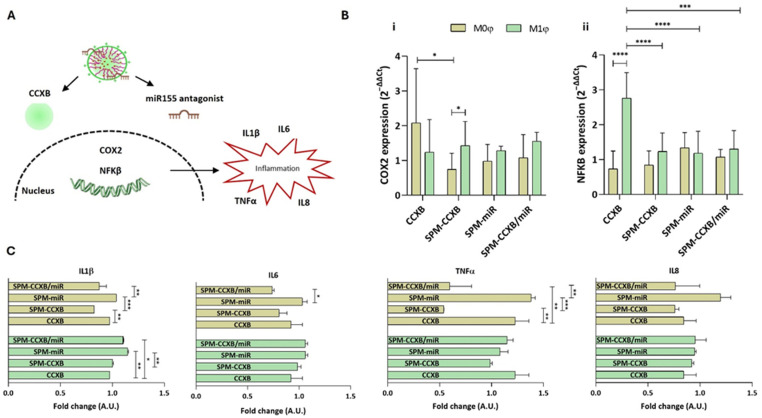
Evaluating SPM-CCXB/miR significance over Mφ behavior. (**A**) Schematic showing Mφ inflammatory cascade associated with CCXB and miR155 antagonist. (**B**) Gene expression analysis of (i) *COX2 (aka PTGS2)* and (ii) *NF*κ*B*, 24 h after CCXB and SPM-based delivery to M0φ or M1φ. The expression was normalized against *GAPDH* housekeeping gene, and the quantification was performed according to the Livak method, considering the control condition (untreated M0φ or M1φ) as calibrator. Data were analyzed by the Two-Way ANOVA, followed by the Sidak test (* *p* < 0.05, *** *p* < 0.001, **** *p* < 0.0001). (**C**) Multiplex immunoassay for the detection of inflammatory cytokines. Two-fold change transformations were performed by dividing the mean value of the cytokine quantified by the mean of the controls: untreated M0φ or M1φ, and M0φ or M1φ treated with SPM-based systems. Data were analyzed by the Two-Way ANOVA, followed by the Tukey test (* *p* < 0.05, ** *p* < 0.01 and *** *p* < 0.001). Data represent the mean ± SD of at least three independent experiments.

Since early diagnosis is difficult, effective treatments for chronic inflammation diseases would greatly benefit from a regulated inflammatory response, along with a reinforcement of the self-resolving mechanisms. Controlling M1φ inhibits inflammatory factors and the progression of damage, while influencing M0φ ensures that the inflammatory response does not escalate unnecessarily, reducing the risk of chronic conditions. Thus, we studied the significance of SPMs loaded with both medicines in modulating Mφ function on both naïve and inflammatory Mφ populations. We hypothesized that the Mφ interactions with CCXB and miR155 antagonist integrates a regulatory network that controls the inflammatory cascade (Figure 5A), which justifies the therapeutic combination of CCXB and the miR155 antagonist. CCXB is a well-known COX2 suppressor, which leads to the mitigation of inflammatory mediators. Additionally, NFκB is a key regulator of COX2 production [46,47] and the miR155 depends on NFκB.

Elevated miR155 levels prolong the inflammatory response by targeting negative regulators of inflammation, thereby sustaining NFκB activity. The inhibition of the miR155/NFκB signaling pathway was reported by Liu et al. to significantly weaken the expression of inflammatory cytokines in neonatal pig acute respiratory distress syndrome [48]. Furthermore, Zhang et al. demonstrated that miR155 knockdown improved nerve function in cerebral ischemia-reperfusion injury by restraining inflammation [49].

Our results demonstrated that the SPM treatments in M1φ led to *COX2* levels very similar to the ones observed with the free form of CCXB (*p* > 0.05) (Figure 5B(i)). Unlike M1φ, M0φ seem to be more responsive to the SPM treatment, evidencing a more pronounced decrease in *COX2* expression (*p* < 0.05). Interestingly, this tendency reverts for the *NFκB* where a significant decrease is observed in M1φ treated with SPM formulations (Figure 5B(ii)).

Regarding the cytokine’s secretion, no differences were observed between free-CCXB and SPM-CCXB in M1φ. On the other hand, the effect of SPM-CCXB and dual-loaded SPM in M0φ is evident, specifically for the IL1β and TNFα (*p* < 0.05 in comparison to free CCXB) (Figure 5C). IL1β is a potent inflammatory mediator leading to the activation of multiple signaling cascades including NFκB and mitogen-activated protein kinase (MAPK) pathways, which further regulate inflammatory cytokine expression (e.g., IL6, TNFα, and MCP1), tissue remodeling, and immune cell activation.

The distinct responses between M0φ and M1φ may be explained by Mφ functional plasticity to adapt and to manage signals from their microenvironment, resulting in differences in receptor expression, intracellular signaling pathways, gene expression profiles, functional specialization, and feedback mechanisms.

In the case of SPMs and the selected cargos, naïve Mφ inherently have lower baseline levels of cytokine expression compared to M1φ, as we could observe in the blot analysis from the previous figure. The introduction of the nanoplatforms might more effectively suppress pro-inflammatory expression in M0φ, as there are fewer inflammatory stimuli to counteract the treatment’s effects. The heightened activation state and the type of signaling cascade could render M1φ less responsive to the cargo amounts and SPM composition, as their state is maintained by reinforcing pro-inflammatory signals. For instance, NFκB is activated either by a canonical or non-canonical manner [50] depending on the Mφ type, which may contribute to idiosyncratic susceptibility to inflammatory mediators and/or therapeutic agents.

Since M0φ are more influenced by the SPM treatment, the Mφ functional state and their population density stand as critical parameters to account for in precision cell therapies. Directing M0φ macrophages can help halt their functions into pro-inflammatory Mφ and influence their differentiation towards anti-inflammatory M2 phenotypes, improving treatment options. These outcomes can open new possibilities for precision therapies, but further research is necessary to understand SPM impact and cargo effects in different immune cells.

## 3. Conclusions

This study provides insights on the interdependency of anti-inflammatory agents exerting a fine-tuned regulatory action over naïve and classically activated Mφ to counteract the limitations of anti-inflammatory drugs and fulfill the need for improved and safe therapeutics to resolve chronic inflammation. As most of the reported works address hydrophobic drugs alone, stored in the micellar core, this work also contributed to unlocking the potential of the micelle shell to incorporate hydrophilic therapeutic molecules. The SPMs efficiently carried both traditional drugs and miR, ensuring their stability, bioavailability and therapeutic action to drive Mφ functional profiles. The system is viable and takes advantage of using parts that have already proven clinical benefits, such as SPIONs for imaging technologies, CCXB as a primary treatment for different diseases, and miR as an advanced therapy for cancer. The SPM components’ clinical compliance, non-hemolytic properties, and stability in physiologically compatible fluids are crucial factors for predicting the safety, local, and systemic efficacy of SPMs as medical devices in human therapy.

In vitro studies showed that CCXB and CCXB-miR loads were more efficient in reducing the genetic expression of COX2 and NFκB in Mφ in comparison to free forms of CCXB, suggesting an SPM value for a safer and more effective delivery of therapeutic agents, especially the ones oriented to immune cells.

The co-delivery of CCXB and miR155 antagonist had a synergistic effect on the inflammatory profile of Mφ. miRs can complement the action of anti-inflammatory drugs by targeting additional pathways that the drugs alone might not access to benefiting from a precision action and lower dosages while potentially minimizing the side effects associated with broad-spectrum anti-inflammatory drugs.

Interestingly, naïve Mφ seem to be more susceptible to dual-loaded SPMs, which can provide a strategic point of intervention in the immune response including blocking M1 activation in inflammation-associated pathologies. Such an approach is particularly appreciated in treating chronic inflammatory conditions, autoimmune diseases, and in post-surgical healing, where managing the balance between pro- and anti- inflammatory paths is crucial for recovery and long-term health.

These findings further highlight the modeling potential of this nanoplatform for a more comprehensive and effective treatment for chronic inflammatory diseases enabling precision and personalized medicine approaches.

## 4. Materials and Methods

### 4.1. Production of SPM and CCXB-Loaded SPM

Chitosan *grafted* with palmitic acid (PA-*g*-CHI) was synthetized according to a previously reported procedure [51] and was dissolved in a 20 mM acetic acid (Honeywell Fluka, Fluka, Germany) solution at 1 mg/mL and the pH of this solution was adjusted to 6.5 with NaOH (99.0%, PanReac QUIMICA S.L.U., Castellar del Vallés, Barcelona, Spain) 5 M. A volume of 200 µL of SPIONs (10 nm size, 5 mg/mL in chloroform, Sigma-Aldrich, St. Louis, MO, USA) was added to 5 mL of the PA-*g*-CHI solution to obtain a 1:5 SPION:PA-*g*-CHI mass ratio. SPMs were prepared by an ultrasonication method. The mixture was ultra-sonicated in an ice-cooled bath for 20 min and stirred overnight at room temperature (RT) to evaporate the remaining chloroform. The resulting suspension was dialyzed (dialysis devices, ready-to-use, Float-A-Lyzer G2, 300 kD, 2–5 mL, Sigma-Aldrich, St. Louis, MO, USA) for 2 days with frequent changes of water. CCXB (98.1%, Sigma-Aldrich) was also incorporated in the core of the SPMs using two SPION:PA-*g*-CHI:CCXB mass ratios: 1:5:0.1 and 1:5:0.2. The procedure used was the same as for the SPM production except for initially adding CCXB dissolved in chloroform (99.4%, Honeywell Riedel-de Haën, Seelze, Germany) (100 µL and 200 µL) to the PA-*g*-CHI solution.

#### 4.1.1. Determination of the Size and Charge of the SPM-Based Systems

The hydrodynamic diameter, size distribution, and surface charge of the nanoparticles were measured in a Malvern NanoZS (Malvern Instruments, Worcestershire, UK) equipped with a 4 mW He-Ne laser operating at 633 nm. The scattered light was detected at a back-scatter angle (173°). For dynamic light scattering (DLS) and electrophoretic light scattering (ELS) measurements, the nanoparticles were suspended in an acidic solution at a concentration of about 0.1 mg/mL and filtered through a 0.45 µm PES syringe filter. A volume of 1 mL of each sample was placed in a disposable polystyrene cuvette (DLS) or folded Capillary cell (ELS) and 3 measurements were performed for each sample.

#### 4.1.2. Fluorescence Emission Spectrum as Function of Concentration of SPM-CCXB

Solutions of SPM-CCXB were prepared in an acetic acid solution with different concentrations (0–1000 µg/mL) and the fluorescence emission spectra were recorded (500–700 nm) in a microplate reader (Varioskan Lux, Thermo Scientific, Waltham, MA, USA) using an excitation wavelength of 471 nm.

#### 4.1.3. Determination of SPION-Loading Efficiency and Content in SPM and SPM-CCXB

The iron (Fe) concentration in SPMs and SPM-CCXB was determined by inductively coupled plasma-optical emission spectroscopy (ICP-OES) using a HORIBA JOBIN YVON, Inc ICP spectrometer (Palaiseau, France).

Concentrated nitric acid (69% for analysis EMSURE^®^ ACS, Reag. Ph Eur, Merck, Darmstadt, Germany) was added to the samples (SPM and SPM-CCXB) and heated at 65 °C for 1 h. After this time, the solution was allowed to cool to RT and subsequently diluted with ultrapure water to 5% HNO_3_. A calibration curve was performed before sample analysis using eight Fe standard samples (0–10 mg/L). These were prepared by diluting a Fe standard solution (1000 mg/L Fe in 5% HNO_3_, Alfa Aesar, Kandel, Germany) with ultrapure water. The Fe concentration was determined at 259.94 nm.

#### 4.1.4. Determination of CCXB-Loading Efficiency and Content

To determine the loading efficiency (LE) and loading content (LC) of the incorporated drug, CCXB-loaded micelles (500 μL) were lyophilized. Dried particles were dissolved in DMSO which allowed for the disruption of the micellar structure, followed by vortexing and centrifugation. The CCXB concentration in each sample was quantified by fluorescence spectrometry (Varioskan Lux, Thermo Scientific) using an excitation wavelength of 471 nm and emission wavelength of 550 nm (free CCXB). Calibration curves with CCXB dissolved in DMSO were also performed (0–50 μg/mL).

#### 4.1.5. Characterization of the Release Kinetics of CCXB from SPM

To assess the release behavior of CCXB from CCXB-loaded SPMs, 100 μL of CCXB-loaded SPMs was incubated with PBS pH 7.4 or acetic acid solution 20 mM pH 5.2 (AA) at 37 °C in a black 96-well plate (Costar^®^, VWR, Corning, NY, USA). The assay was performed in a microplate reader (Varioskan Lux, Thermo Scientific, Waltham, MA, USA) using an excitation wavelength of 471 nm and an emission wavelength of 586 nm (drug-loaded SPM), and the mixture was shaken for 5 s before each reading. Immediately after the incubation, the fluorescence intensity was periodically recorded for up to 11 h.

### 4.2. Production of SPM-miRs

SPM-miRs were produced using commercial antagonist hsa-miR-155-5p (Dharmacon (Lafayette, CO, USA), mature sequence: UUAAUGCUAAUCGUGAUAGGGGU) diluted with RNase-free water (Thermo Scientific, Waltham, MA, USA) to a final concentration of 5 µM. For the experiments, 0.75 µg of SPM was loaded with 0.15 µg of an miR cargo. For that, SPMs were incubated with miR155 antagonist for 30 min at room temperature to allow the formation of the SPM-miR.

### 4.3. Production and Colloidal Stability Assessment of SPM-CCXB/miR

SPM-CCXB/miR particles were produced in two stages. In the first stage, SPM-CCXB particles were produced as described in Section 4.1. Then, the negatively charged miR molecules were electrostatically bonded to the positively charged CHI from SPM-CCXB particles forming the dual-loading SPM. The mass proportions of SPM-CCXB/miR were the same as described in previous sections.

For evaluation of the colloidal stability of SPM-CCXB/miR in physiological media, we have also performed DLS and ELS measurements of SPM-CCXB/miR in PBS and simulated body fluid (SBF, prepared according to [52]). For that, SPM-CCXB/miRs were diluted in PBS 7.4 and SBF to a final concentration of 0.1 mg/mL and filtered through a 0.45 µm PES syringe filter.

### 4.4. Macrophage Culture and Differentiation

Human monocytes (THP1, American Type Culture Collection, Manassas, VA, USA) were cultured in Roswell Park Memorial Institute Medium (RPMI, Sigma-Aldrich, St. Louis, MO, USA) supplemented with 1% L-glutamine (Thermo Scientific, Waltham, MA, USA) and 10% fetal bovine serum (FBS) (Thermo Scientific, Waltham, MA, USA) in a humidified 5% CO_2_ atmosphere, differentiated into naïve macrophages (M0φ) and subsequently polarized to M1φ. Briefly, monocytes were differentiated into M0φ with 100 nM phorbol 12-myristate 13-acetate (PMA, Sigma-Aldrich, St. Louis, MO, USA) for 24 h, followed by a 24 h rest period in the described culture conditions. For M1φ polarization, cells were further incubated with LPS (100 ng/mL, Sigma-Aldrich, St. Louis, MO, USA) and IFNγ (20 ng/mL, PeproTech, Rocky Hill, NJ, USA) for 24 h.

### 4.5. Assessment of Cell Uptake of Free-Form and SPM-Loaded CCXB

To investigate the internalization of celecoxib-NBD, PMA-differentiated THP1 were cultured in 24 well plates (2 × 10^5^ cells/well, Corning Inc., New York, NY, USA) before incubation with a concentration gradient of celecoxib-NBD (0, 2.5, 5.0, and 7.3 µg/mL, which correspond to 0, 4.3, 8.6, and 12.4 µM, respectively) and with two formulations of SPM loaded with CCXB SPM-CCXB (0.1) and SPM-CCXB (0.2). These concentrations were selected based on the literature reports [53] and validated by a study performed by our group in order to identify physiologically compatible drug ranges for the type of cells and cellular densities studied.

The cell uptake of SPM-based systems for this and other biological assays was performed using the magnetofection protocol or by gravity (non-magnetic field actuation). In the case of magnetofection, upon adding the SPM-based systems to the Mφ (100 µL/well), cell-cultured plates were exposed to a stationary magnetic field for 20 min (350 mT/well), following the protocols previous established by our group [21], using a magnefect nano device (nanoTherics Ltd., Warrington, UK)

Afterwards, cells were collected using mild mechanical detachment, resuspended in acquisition buffer (1% formaldehyde in PBS), and acquired in a FACS Calibur equipment (BD Biosciences, Berkshire, UK). The cells were identified using forward and side scattering analysis. A minimum of 20,000 cells were acquired and data analysis on FL1 was performed using the Cell Quest software. Acquired data were normalized with untreated cells. Also, the cultured Mφ with free-form celecoxib and SPM-CCXB were observed under an inverted fluorescence microscope with a THUNDER Imaging System (DMi8, Leica, Wetzlar, Germany).

In the case of the naïve and M1-primed macrophages treated with SPM-CCXB/miR, cell uptake was confirmed by intracellular celecoxib fluorescence measurements. For that, cells treated with SPM-based systems were washed with PBS. Before the reading of the CCXB fluorescence at 471/550 nm, fresh PBS was added to the wells. The outcomes were compared to the ones of SPMs (non-fluorescent) and SPM-CCXB (fluorescent due to the presence of NBD).

### 4.6. Cell Viability Evaluation of SPM-Based Systems

The viability assay was performed in macrophages treated with the different SPM-based particles, namely SPM, SPM-CCXB, SPM-miR, and SPM-CCXB/miR. The cell metabolic activity was assessed by PrestoBlue™ reagent (Thermo Scientific, Waltham, MA, USA). Briefly, 1/10th volume of the reagent was directly added to the cells in culture medium and incubated at 37 °C, protected from light. The results of fluorescence were recorded in a microplate reader (Varioskan Lux, Thermo Scientific, Waltham, MA, USA) at 560/590 nm. Untreated cells were used as the positive control.

### 4.7. Hemolytic Assay

The hemolytic properties of SPMs, SPM-CCXB, and SPM-miR were assessed according to the protocol recommended by the International Organization for Standardization: ISO 10993-4:2017, involving contact with red blood cells (RBC). Human RBC concentrates from three different donors were obtained from the Portuguese Institute for Blood and Transplantation (IPST), adhering to the ethical guidelines and with donor consent. Platelet-rich plasma and the buffy coat were discarded, and the RBCs were washed 2 times with PBS. After each washing, a centrifugation for 5 min at 3700 rpm was performed. The RBC concentrate was diluted to a final concentration of 5 vol%. The 15, 30, or 50 µg/mL SPM suspensions, SPM-CCXB, and SPM-miR (100 µL) were added to 1000 μL of the RBC suspension. PBS and 1% Sodium Dodecyl Sulfate (SDS) were used as negative and positive controls, respectively. After a 1 h incubation at 37 °C under mild agitation (200 rpm), samples were washed with PBS and centrifuged at 120 g for 10 min. The absorbance of the obtained supernatants was measured at 540 nm in a microplate reader (Varioskan Lux, Thermo Scientific, Waltham, MA, USA). To determine the hemolytic ratio, the following equation was used:

Hemolytic ratio = (sample—negative control)/(positive control–negative control)

The hemolysis ratio was interpreted as follows: inferior to 2%, non-hemolytic; between 2–5% slightly hemolytic; and higher than 5%, hemolytic samples.

### 4.8. Cytokine Array

Mφ were cultured in 24 wells plates (2 × 10^5^ cells/well, Corning Inc., New York, NY, USA), polarized into M0φ or M1φ as previously described, and the supernatants were analyzed using a Human Cytokine Array (RayBiotech, Inc., Norcross, GA, USA) following the manufacturer’s instructions. RPMI cell culture medium was used as control.

### 4.9. RNA Extraction and qPCR

RNA was extracted using mini columns according to the miRNeasy Mini Kit protocol (Qiagen, Hilden, Germantown, MD, USA). RNA concentration and purity were determined using a spectrophotometer (Nanodrop 2000, Thermo Scientific, Waltham, MA, USA). RNA was converted to cDNA using High-Capacity cDNA Reverse Transcription Kit (Thermo Scientific, Waltham, MA, USA) according to the manufacturer’s instructions. Quantitative polymerase chain reaction (qPCR) was performed using PowerUp SYBR Green Master Mix (Thermo Scientific, Waltham, MA, USA). Each sample was run in triplicate and normalized to endogenous control glyceraldehyde 3-phosphate dehydrogenase (*GAPDH*). Data represent fold change from untreated cells (2^−ΔΔCT^). Primer (Integrated DNA Technologies (Coralville, IA, USA) or Invitrogen (Carlsbad, CA, USA)) sequences can be found in Table S1 (Supplementary Information).

### 4.10. Multiplex Immunoassays

The cytokine secretion of M0φ or M1φ, after the drug and SPM-based treatments, was determined by a 10-Plex human cytokine assay (Thermo Scientific, Waltham, MA, USA) and read on a Luminex MAGPIX system. Two-fold change transformation was performed by dividing the mean value of the cytokine quantified by the mean of the controls: untreated M0φ or M1φ and treated M0φ or M1φ SPMs. The SPM treatments were used as follows: (i) free SPM (SPM), (ii) loaded with CCXB (SPM-CCXB), (iii) loaded with miR155 antagonist (Dharmacon, Lafayette, CO, USA) (SPM-miR), (iv) double loaded (SPM-CCXB/miR), and (v) drug only (CCXB).

### 4.11. Statistical Analysis

Results are expressed as the mean ± standard deviation (SD), representative of at least three independent experiments. One-Way Analysis of Variance (ANOVA) with Dunnett’s post hoc test was used for multiple comparisons in the hemolysis experiments, whereas for the qPCR and multiplex immunoassay, Two-Way ANOVA with Sidak’s or Tukey’s post hoc test was used. Statistical analysis was performed using Prism GraphPad 8.01 software. * *p* < 0.05 represents the threshold for statistical significance.

## Figures and Tables

**Table 1:** SPION- and drug-loading efficiency (LE) and loading content (LC).

	SPION		CCXB	
	LE (%)	LC (%)	LE (%)	LC (%)
SPM	76 ± 7	15 ± 2	-	-
SPM-CCXB (0.2)	71 ± 5	9.6 ± 0.7	42 ± 4	16 ± 3

## Data Availability

Dataset available on request from the authors, dataset_file V.1.

## Supplementary Materials

The following supporting information can be downloaded at: https://www.mdpi.com/article/10.3390/ijms251910693/s1.
