# Supplementary material for: Using Hybrid Nanoplatforms to Combine Traditional Anti-Inflammatory Drug Delivery with RNA-Based Therapeutics for Macrophage Reprograming
Source: Int J Mol Sci. 2024 Oct 4;25(19):10693. doi: 10.3390/ijms251910693 (PMC11476774; doi:10.3390/ijms251910693)
Supplement: Supplementary file 1 [file ijms-25-10693-s001.zip › ijms-3166164-supplementary.pdf]

SUPPLEMENTARY INFORMATION

Using Hybrid Nanoplatforms to Combine Traditional Anti-inflammatory Drug Delivery with RNA-based Therapeutics for Macrophage Reprograming

Ana F. Almeida[1,2], Margarida S. Miranda[1,2], Rui L. Reis[1,2], Manuela E. Gomes[1,2*], Márcia T. Rodrigues[1,2*]

[1] 3B's Research Group, I3Bs - Research Institute on Biomaterials, Biodegradables and Biomimetics, University of Minho, Headquarters of the European Institute of Excellence on Tissue Engineering and Regenerative Medicine, AvePark, Parque de Ciência e Tecnologia, Zona Industrial da Gandra, 4805-017 Barco, Guimarães, Portugal

[2] ICVS/3B's - PT Government Associate Laboratory, Braga/Guimarães, Portugal

*Correspondence:

M.T. R, mrodrigues@i3bs.uminho.pt ; Tel.: +351 253 510 947

M. E. G, megomes@i3bs.uminho.pt; Tel.: +351 253 510 904

Figure S1. SPM colloidal stability in acidic aqueous suspension.

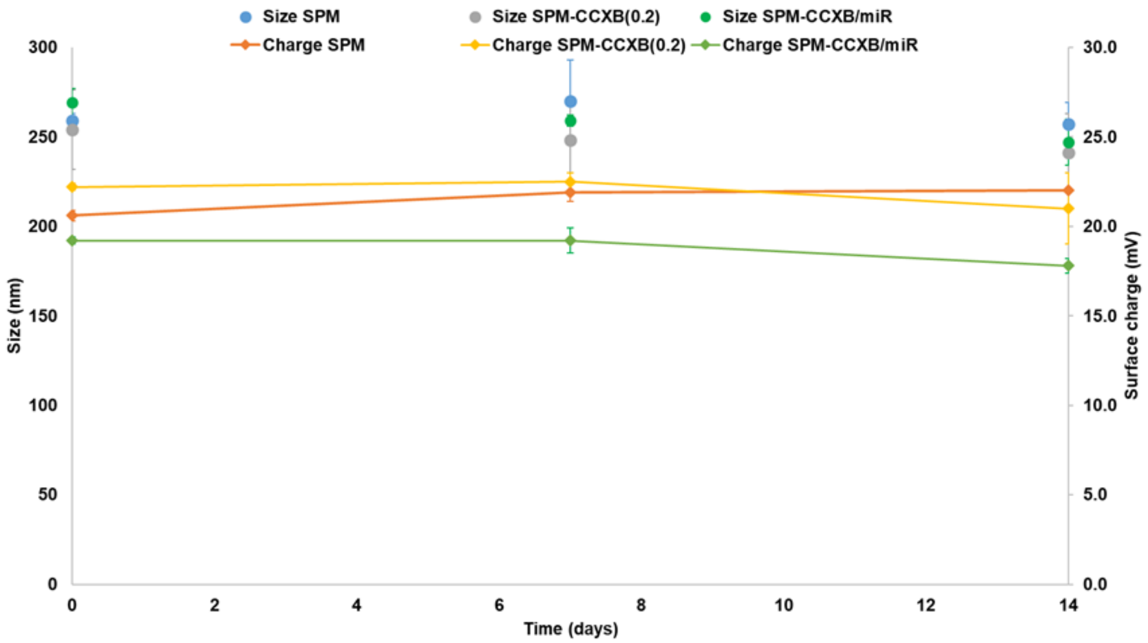

Hydrodynamic size, and surface charge of SPM, SPM-CCXB(0.2) and SPM-CCXB/miR in acidic aqueous solution for 15 days and storage at 4ºC.

Table S1. Primers sequences used in *q*PCR.

GAPDH	F: GTAACCCTGTGAACCCCATT<br>R: CCATCCAATCGGTAGTAGCG
COX2 (aka PTGS2)	F – ATGGGGTGATGAGCAGTTGT<br>R – GAAAGGTGTCAGGCAGAAGG
NFκB	F: GAAGCACGAATGACAGAGGC<br>R: GCTTGGCGGATTAGCTCTTTT
